# Diabetic Foot Ulcers: Pathophysiology, Immune Dysregulation, and Emerging Therapeutic Strategies

**DOI:** 10.3390/biomedicines13051076

**Published:** 2025-04-29

**Authors:** John Dawi, Kevin Tumanyan, Kirakos Tomas, Yura Misakyan, Areg Gargaloyan, Edgar Gonzalez, Mary Hammi, Serly Tomas, Vishwanath Venketaraman

**Affiliations:** 1 College of Osteopathic Medicine of the Pacific, Western University of Health Sciences, Pomona, CA 91766, USA; john.dawi@westernu.edu (J.D.); yura.misakyan@westernu.edu (Y.M.); areg.gargaloyan@westernu.edu (A.G.); edgar.gonzalez@westernu.edu (E.G.); mary.hammi@westernu.edu (M.H.); 2 College of Podiatric Medicine, Western University of Health Sciences, Pomona, CA 91766, USA; kevin.tumanyan@westernu.edu (K.T.); kirakos.tomas@westernu.edu (K.T.); 3 Oakland University William Beaumont School of Medicine, Rochester, MI 48309, USA; stomas@oakland.edu

**Keywords:** advanced glycation end products (AGEs), receptor for AGEs (RAGE) activation, macrophage polarization (M1/M2 imbalance), vascular endothelial growth factor (VEGF) therapy, negative pressure wound therapy (NPWT)

## Abstract

Diabetic foot ulcers (DFUs) are among the most common and debilitating complications of diabetes mellitus (DM), affecting approximately 15–25% of patients and contributing to over 85% of non-traumatic amputations. DFUs impose a substantial clinical and economic burden due to high recurrence rates, prolonged wound care, and frequent hospitalizations, accounting for billions in healthcare costs worldwide. The multifactorial pathophysiology of DFUs involves peripheral neuropathy, peripheral arterial disease, chronic inflammation, and impaired tissue regeneration. Recent studies underscore the importance of immune dysregulation—specifically macrophage polarization imbalance, regulatory T cell dysfunction, and neutrophil impairment—as central mechanisms in wound chronicity. These immune disruptions sustain a pro-inflammatory environment dominated by cytokines, such as TNF-α, IL-1β, and IL-6, which impair angiogenesis and delay repair. This review provides an updated synthesis of DFU pathogenesis, emphasizing immune dysfunction and its therapeutic implications. We examine emerging strategies in immunomodulation, regenerative medicine, and AI-based wound technologies, including SGLT2 inhibitors, biologics, stem cell therapies, and smart dressing systems. These approaches hold promise for accelerating healing, reducing amputation risk, and personalizing future DFU care.

## 1. Introduction

Diabetes mellitus (DM) remains a global health burden, with the World Health Organization (WHO) projecting that the prevalence of diabetes will rise to approximately 700 million individuals by 2045 [1]. Type 2 diabetes mellitus (T2DM), which comprises the vast majority of diabetes cases globally, is defined by insulin resistance and a relative insulin deficiency [2]. The pathophysiology of diabetes is multifactorial, but its hallmark is chronic hyperglycemia, which leads to a cascade of complications that affect multiple organs, most notably the microvasculature [3]. Diabetic microvascular complications, including diabetic retinopathy, nephropathy, neuropathy, and diabetic foot ulcers (DFUs), are some of the leading causes of morbidity and disability in patients with diabetes [4]. Diabetic foot ulcers are a common and severe complication of T2DM, affecting between 19% and 34% of individuals with diabetes during their lifetime [5]. DFUs significantly impair quality of life and are often associated with high rates of infection, hospitalization, and amputations [6]. The development of DFUs is closely linked to both microvascular and macrovascular complications, including diabetic neuropathy and peripheral artery disease (PAD), both of which are significantly more prevalent in individuals with diabetes compared to the general population [7]. Hyperglycemia-induced oxidative stress, endothelial dysfunction, and inflammatory processes contribute to the pathogenesis of these vascular complications, promoting the progression of ulcers and delaying wound healing [8]. In addition to these mechanisms, the global clinical and economic burden of DFUs is substantial, with annual costs exceeding $40 billion and contributing significantly to hospitalization and disability rates [9].

In recent years, there has been increasing recognition of the role of immune dysregulation in the development and persistence of diabetic foot ulcers. Chronic inflammation and immune dysfunction impair the healing process of DFUs, leading to persistent infections and tissue damage. Inflammatory mediators, including cytokines and chemokines, contribute to the creation of a pro-inflammatory wound environment, which disrupts normal wound healing and fosters an environment conducive to infection and chronicity [10]. Immune cells, such as neutrophils, macrophages, and T lymphocytes, play pivotal roles in both the initiation and resolution of inflammation. In the context of diabetes, however, the immune response is dysregulated, leading to an impaired healing response and increased susceptibility to infections [11]. Neutrophil dysfunction, regulatory T cell depletion, and impaired angiogenic signaling have been observed in non-healing DFUs and further exacerbate the chronic inflammatory state [12].

Several studies have explored the mechanisms by which hyperglycemia affects immune function, emphasizing the role of oxidative stress and advanced glycation end products (AGEs) in immune cell dysfunction. AGEs, which are formed by the non-enzymatic glycation of proteins, lipids, and nucleic acids under hyperglycemic conditions, have been implicated in endothelial dysfunction and increased vascular permeability, which are crucial steps in the pathogenesis of diabetic microvascular complications [13]. Furthermore, AGEs interact with the receptor for advanced glycation end products (RAGE) on immune cells, leading to the activation of pro-inflammatory pathways that exacerbate tissue injury [14]. The therapeutic management of diabetic foot ulcers requires a comprehensive approach, involving strict glycemic control, optimal wound care, and immune modulation. While glycemic control is critical in preventing and managing DFUs, it is often insufficient on its own. Emerging therapeutic strategies that target immune dysregulation and inflammation have shown promise in improving wound healing and preventing recurrence of DFUs. Pharmacological agents, such as SGLT2 inhibitors and biologics that modulate immune responses, offer new avenues for treatment [15]. Additionally, advanced wound care techniques, including the use of growth factors, bioactive dressings, and stem cell therapies, are being investigated for their ability to enhance tissue repair and regeneration [16].

Technologies such as artificial intelligence (AI)-driven wound image analysis, machine learning algorithms for ulcer classification, and wearable monitoring tools are being evaluated as adjuncts for early detection and individualized DFU management strategies [17]. In this review, we will explore the pathophysiology of diabetic foot ulcers, with a focus on the role of immune dysregulation in wound healing and the development of chronic ulcers. We will also discuss the current therapeutic strategies for managing diabetic foot ulcers, including emerging treatments that target the immune system and inflammatory pathways.

## 2. Pathophysiology of Diabetic Foot Ulcers

Diabetic foot ulcers (DFUs) are the result of a complex interplay of metabolic, vascular, immunological, and mechanical factors that ultimately lead to chronic, non-healing wounds in the lower extremities. The pathogenesis of DFUs is heavily influenced by persistent hyperglycemia, which induces endothelial dysfunction, oxidative stress, chronic inflammation, impaired angiogenesis, and defective immune responses, all of which contribute to delayed wound healing and increased susceptibility to infections [18]. Hyperglycemia also drives metabolic reprogramming in immune cells, contributing to prolonged inflammation, impaired macrophage plasticity, and resistance to tissue remodeling [19].

### 2.1. The Role of Hyperglycemia and Metabolic Dysregulation

Chronic hyperglycemia disrupts multiple cellular pathways, including polyol pathway activation, advanced glycation end product (AGE) formation, and hexosamine pathway dysregulation, all of which impair tissue repair mechanisms [20].

- **Polyol Pathway Activation**: In hyperglycemic conditions, excess glucose is shunted into the polyol pathway, where aldose reductase converts glucose into sorbitol, which is then metabolized into fructose by sorbitol dehydrogenase [21]. The accumulation of sorbitol leads to osmotic stress, depletion of nicotinamide adenine dinucleotide phosphate (NADPH), and reduced glutathione (GSH) availability, exacerbating oxidative stress and damaging endothelial cells [22].
- **AGEs and RAGE Activation**: Hyperglycemia drives the formation of advanced glycation end products (AGEs), which accumulate on matrix proteins such as collagen and laminin. This increases tissue stiffness and disrupts cellular communication critical for healing [17]. AGEs bind to the receptor for advanced glycation end products (RAGE), triggering inflammatory cascades that exacerbate endothelial dysfunction and increase the production of reactive oxygen species (ROS), further contributing to cellular damage and impaired wound healing [23].
- **Hexosamine Pathway Dysregulation**: Excess glucose is also metabolized via the hexosamine biosynthetic pathway, resulting in excessive glycosylation of proteins involved in wound healing, such as growth factors and signaling molecules, impairing their function and further delaying wound repair [24].

### 2.2. Endothelial Dysfunction and Impaired Microvascular Function

Diabetes is associated with significant endothelial dysfunction, which reduces nitric oxide (NO) availability, impairs vasodilation, and decreases capillary perfusion, all of which delay ulcer healing [25]. NO plays a critical role in regulating vascular tone, and its suppression results in increased vascular resistance and reduced oxygen and nutrient delivery to wounded tissues [25]. Hyperglycemia-induced dyslipidemia promotes endothelial damage by increasing the levels of oxidized low-density lipoprotein (oxLDL), which triggers inflammation and atherosclerotic changes within the microvasculature [11].

- **Capillary Basement Membrane Thickening**: Persistent hyperglycemia leads to thickening of the capillary basement membrane due to excessive deposition of AGE-modified extracellular matrix proteins. This impairs the exchange of oxygen and nutrients between blood and tissues, contributing to hypoxia in ulcerated areas [26].
- **Microthrombosis and Impaired Fibrinolysis**: Diabetes promotes a pro-thrombotic state by increasing platelet aggregation and reducing fibrinolytic activity, leading to microvascular occlusions that further impair perfusion in ischemic tissues [27]. Emerging data also suggest that endothelial cell senescence plays a role in impaired angiogenic signaling in DFUs [28].

### 2.3. Neuropathy and Loss of Protective Sensation

Diabetic neuropathy, which affects up to 50% of individuals with diabetes, is one of the primary factors in DFU development [15]. This condition is characterized by sensory, motor, and autonomic dysfunction, all of which contribute to ulcer formation and chronicity.

- **Sensory Neuropathy and Loss of Pain Perception**: Damage to small nerve fibers results in loss of pain, temperature, and pressure sensation, leading to unnoticed trauma, microabrasions, and repetitive pressure injuries that contribute to ulcer formation [29].
- **Motor Neuropathy and Biomechanical Abnormalities**: Damage to motor neurons leads to muscle atrophy and imbalance, resulting in foot deformities such as claw toes, hammertoes, and Charcot foot. These deformities alter the pressure distribution on the plantar surface of the foot, causing areas of excessive pressure that predispose patients to ulceration [30].
- **Autonomic Neuropathy and Skin Integrity**: Autonomic dysfunction leads to anhidrosis (loss of sweating), resulting in excessively dry, cracked skin that serves as an entry point for bacterial infections. Additionally, arteriovenous shunting impairs blood flow regulation, further compromising tissue perfusion and wound healing [31].

### 2.4. Oxidative Stress and Chronic Inflammation

Hyperglycemia-induced oxidative stress is a major contributor to endothelial dysfunction and delayed wound healing in DFUs [24]. The overproduction of ROS leads to the following:

- **Lipid Peroxidation and DNA Damage**: ROS-induced lipid peroxidation disrupts cell membranes, while DNA damage impairs cellular repair processes [32].
- **Inflammatory Cytokine Activation**: Hyperglycemia triggers the activation of nuclear factor-kappa B (NF-κB), leading to the overproduction of pro-inflammatory cytokines, such as TNF-α, IL-6, and IL-1β, which perpetuate chronic inflammation and inhibit wound healing [33].

The persistent inflammatory environment in DFUs is characterized by a prolonged presence of M1 macrophages, which produce high levels of pro-inflammatory cytokines and matrix metalloproteinases (MMPs), leading to excessive degradation of extracellular matrix components essential for wound repair [34]. Inadequate transition to M2 macrophages and defective resolution pathways contribute further to matrix instability and impaired granulation [35].

### 2.5. Impaired Angiogenesis and Delayed Tissue Repair

Effective wound healing requires angiogenesis, the formation of new blood vessels. However, diabetes disrupts this process through multiple mechanisms, as follows:

- **Reduced VEGF Expression**: Vascular endothelial growth factor (VEGF) is essential for new capillary formation, but hyperglycemia reduces its expression, impairing angiogenesis and delaying tissue regeneration [34].
- **Endothelial Progenitor Cell Dysfunction**: Diabetes reduces the number and function of endothelial progenitor cells (EPCs), which are critical for vascular repair and regeneration, leading to poor wound healing outcomes [36]. Therapies aimed at restoring VEGF signaling and mobilizing EPCs are under investigation in experimental DFU models [36].

### 2.6. Immune Dysfunction and Increased Susceptibility to Infection

Diabetic wounds exhibit profound immune dysfunction, making them highly susceptible to bacterial infections. Diabetes impairs neutrophil chemotaxis and phagocytosis, reducing bacterial clearance and heightening the risk of wound colonization [37]. Additionally, hyperglycemia impairs T cell function, reducing the production of growth factors necessary for wound healing [38]. Recent studies have shown that regulatory T cell suppression and delayed neutrophil apoptosis further prolong inflammation and increase tissue necrosis in DFUs [39].

**Figure 1:**
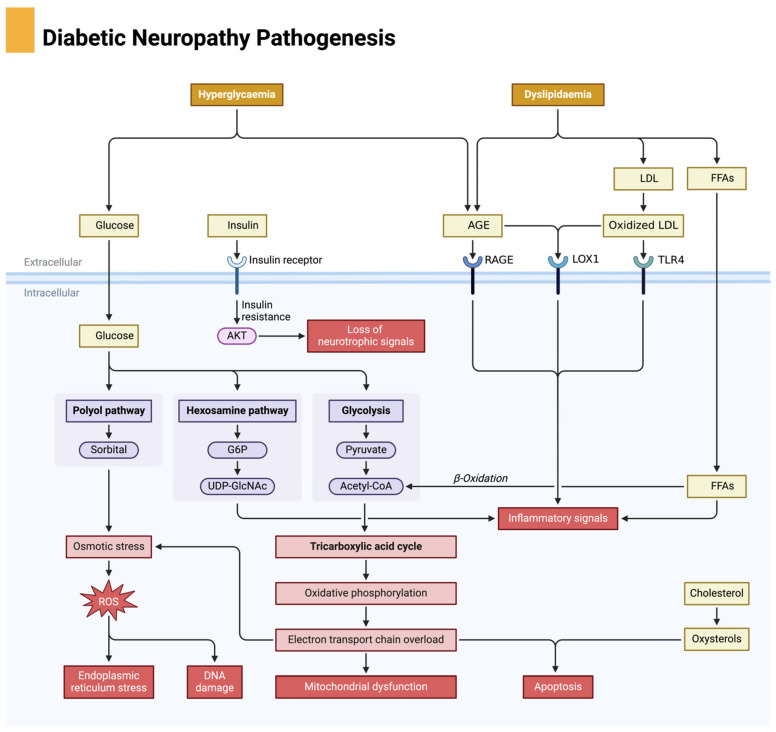
Pathophysiological mechanisms of diabetic neuropathy. Chronic hyperglycemia activates damaging metabolic pathways, including the polyol pathway, advanced glycation end product (AGE) formation, and protein kinase C (PKC) signaling, all of which lead to increased oxidative stress and inflammation. These processes impair axonal transport, blood flow to neurons, and mitochondrial function, ultimately resulting in nerve degeneration and apoptosis. Neuropathy contributes to sensory loss, foot deformities, and increased risk for ulceration. Created in BioRender. Dawi, J. (2025). https://BioRender.com/p83i411 (accessed on 1 February 2025).

The pathophysiology of DFUs is a multifactorial process involving metabolic dysregulation, neuropathy, vascular insufficiency, oxidative stress, chronic inflammation, impaired angiogenesis, and immune dysfunction. Each of these factors creates a self-perpetuating cycle of tissue damage, poor perfusion, and delayed healing, making DFUs one of the most challenging complications of diabetes. Understanding these intricate mechanisms is essential for the development of targeted therapies aimed at breaking this pathological cycle and promoting effective ulcer healing. Diabetic neuropathy is a key contributor to the development of diabetic foot ulcers, driven by metabolic disturbances such as hyperglycemia and dyslipidemia, leading to oxidative stress and nerve damage, as illustrated in Figure 1.

## 3. Immune Dysregulation in Diabetic Foot Ulcer Healing

The immune system plays a critical role in wound healing, involving a tightly regulated series of events, including inflammation, proliferation, and tissue remodeling. These processes are often disrupted in diabetes, leading to delayed wound healing and the development of chronic diabetic foot ulcers (DFUs) [40]. In a healthy individual, the immune response to injury is initially driven by the activation of innate immune cells, such as neutrophils and macrophages. These cells are pivotal in clearing pathogens, managing inflammation, and promoting tissue repair. Neutrophils are the first responders to injury, where they engage in phagocytosis of pathogens and debris and release cytokines and antimicrobial peptides to control infection. Subsequently, macrophages are recruited to the wound site, where they not only clear apoptotic cells and pathogens but also secrete various growth factors that regulate tissue repair.

**Figure 2:**
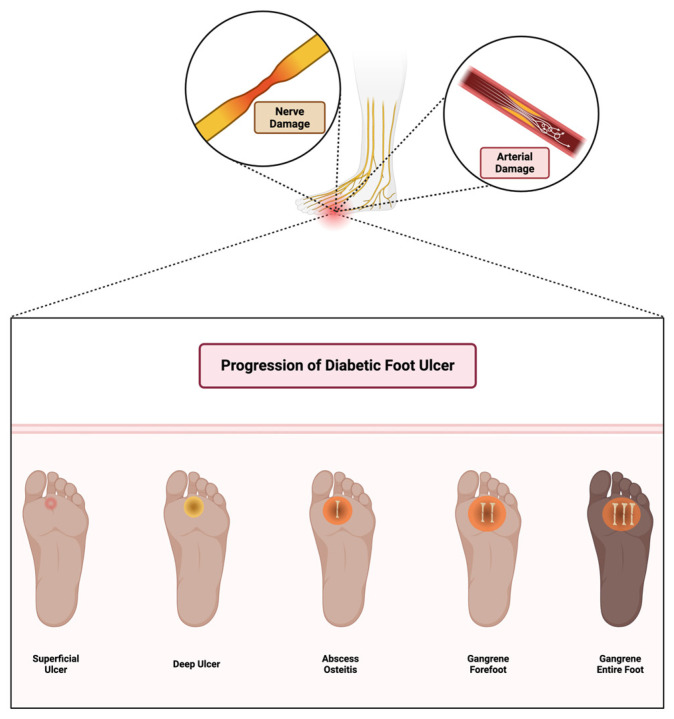
Stages of diabetic foot ulcer progression. Initial damage from peripheral neuropathy leads to loss of protective sensation, abnormal pressure distribution, and unnoticed trauma. Concomitant arterial insufficiency exacerbates tissue ischemia. If left untreated, wounds become infected, progress to deeper tissue involvement and osteomyelitis, and may ultimately require limb amputation. Early detection, offloading, and vascular assessment are critical in preventing progression. Created in BioRender. Dawi, J. (2025). https://BioRender.com/c12c575 (accessed on 1 February 2025).

The immune response in wound healing follows a dynamic pattern: inflammation, resolution, and tissue regeneration. In a typical immune response, the inflammatory phase is transient, resolving as healing proceeds. However, in diabetes, chronic hyperglycemia and subsequent immune dysregulation lead to a prolonged inflammatory response, preventing the transition to the proliferative phase and contributing to the formation of chronic wounds [41]. Diabetic foot ulcers progress through multiple stages, starting from superficial skin lesions and advancing to deep infections, osteitis, and gangrene due to combined neuropathic and vascular impairments, as illustrated in Figure 2.

### 3.1. Hyperglycemia, Oxidative Stress, and the Role of AGEs

Hyperglycemia plays a central role in immune dysfunction in diabetes. Elevated blood glucose levels lead to the non-enzymatic glycation of proteins, lipids, and nucleic acids, resulting in the accumulation of advanced glycation end products (AGEs). AGEs bind to their receptor, RAGE, expressed on immune cells, endothelial cells, and fibroblasts. RAGE binding activates multiple signaling pathways, notably the NF-κB pathway, which is a key regulator of inflammation. NF-κB activation triggers the expression of pro-inflammatory cytokines, such as TNF-α, IL-1β, and IL-6, which sustain the inflammatory response at the wound site and impede its resolution [42].

AGE–RAGE interactions not only exacerbate inflammation but also contribute to endothelial dysfunction. AGEs alter the function of endothelial cells by impairing nitric oxide production, a molecule essential for vasodilation and angiogenesis. As a result, AGE accumulation inhibits the formation of new blood vessels (angiogenesis), which is crucial for providing oxygen and nutrients to the wound tissue, further hindering healing in diabetic patients [43]. In addition, AGEs exacerbate oxidative stress, which in turn activates additional inflammatory mediators. Reactive oxygen species (ROS) generated by cells under high glucose conditions directly damage tissue, and, in the case of immune cells, ROS modulates the activation of signaling cascades such as the MAPK (mitogen-activated protein kinase) pathway, which also contributes to the inflammatory cycle. Furthermore, oxidative stress impairs the phagocytic ability of neutrophils and macrophages, reducing their capacity to clear pathogens and debris effectively. Thus, chronic oxidative stress in diabetes leads to a self-perpetuating cycle of inflammation and impaired immune function that substantially delays wound healing [41]. Mitochondrial dysfunction and impaired redox signaling in diabetic immune cells further amplify ROS generation, sustaining an unresolved inflammatory state. Specifically, dysfunctional mitochondria release excessive superoxide and hydrogen peroxide, triggering NLRP3 inflammasome activation and promoting IL-1β secretion. Impaired mitophagy in macrophages leads to the accumulation of damaged mitochondria, further perpetuating inflammation and tissue injury [44].

### 3.2. Macrophage Polarization and Dysfunction

Macrophages are central to the wound healing process and can polarize into different functional states depending on the signals they receive from the microenvironment. Typically, macrophages polarize from the pro-inflammatory M1 phenotype to the reparative M2 phenotype during wound healing. In diabetes, however, this polarization is skewed toward the M1 phenotype, which exacerbates inflammation and inhibits the transition to healing. M1 macrophages secrete high levels of pro-inflammatory cytokines (TNF-α, IL-1β, and IL-6), which sustain inflammation and inhibit tissue repair [45].

The persistent M1 polarization in diabetic wounds is driven by a combination of factors, including hyperglycemia, AGEs, and oxidative stress. AGE–RAGE interaction directly contributes to this skewing of macrophage function by activating NF-κB and MAPK pathways, which sustain the M1 inflammatory profile. Additionally, the prolonged presence of inflammatory mediators from M1 macrophages impedes the transition to the M2 phenotype, which is essential for wound healing. M2 macrophages promote tissue regeneration by secreting anti-inflammatory cytokines like IL-10 and growth factors such as TGF-β, which stimulate collagen deposition, angiogenesis, and tissue remodeling. Without the proper shift to M2 polarization, diabetic wounds remain in a state of chronic inflammation with impaired tissue repair and regeneration [45]. Therapeutic reprogramming of macrophages through biomaterials or exosome-derived factors is emerging as a novel strategy to restore immune balance and support wound healing in diabetic models. Engineered hydrogels and nanocarriers can deliver IL-4, IL-13, or microRNAs that promote M2 polarization. Additionally, mesenchymal stem cell-derived exosomes have shown promise in downregulating pro-inflammatory signaling pathways such as NF-κB and enhancing angiogenesis in diabetic wound environments [46].

### 3.3. T Cell Dysfunction in Diabetic Wound Healing

T cells are critical in the regulation of the immune response in wound healing, and their dysfunction in diabetes contributes to delayed repair. In diabetes, T cells show impaired activation and migration to the wound site. This dysfunction is partly due to elevated blood glucose, which affects T cell signaling and function. Moreover, in diabetic wounds, there is an increase in the pro-inflammatory cytokine IFN-γ (interferon-gamma) produced by T cells, which exacerbates inflammation and interferes with the resolution of the inflammatory phase [47].

Additionally, a specific subset of T cells, the regulatory T cells (Tregs), is essential for controlling inflammation and facilitating wound healing. Tregs secrete anti-inflammatory cytokines, such as IL-10 and TGF-β, which help switch off the inflammatory response and promote tissue repair. However, in diabetes, Tregs are less abundant, and their function is impaired, leading to a failure in resolving inflammation. This persistent inflammation further delays the wound healing process and contributes to the chronicity of DFUs [48]. Studies have also shown that restoring Treg numbers using low-dose IL-2 or adoptive Treg therapy can enhance wound healing outcomes in diabetic murine models. IL-2 selectively expands the Treg population and promotes secretion of IL-10 and TGF-β, while adoptive transfer of ex vivo expanded Tregs into chronic wounds has been shown to improve epithelialization, neovascularization, and collagen remodeling [49].

### 3.4. Increased Susceptibility to Infections

**Figure 3:**
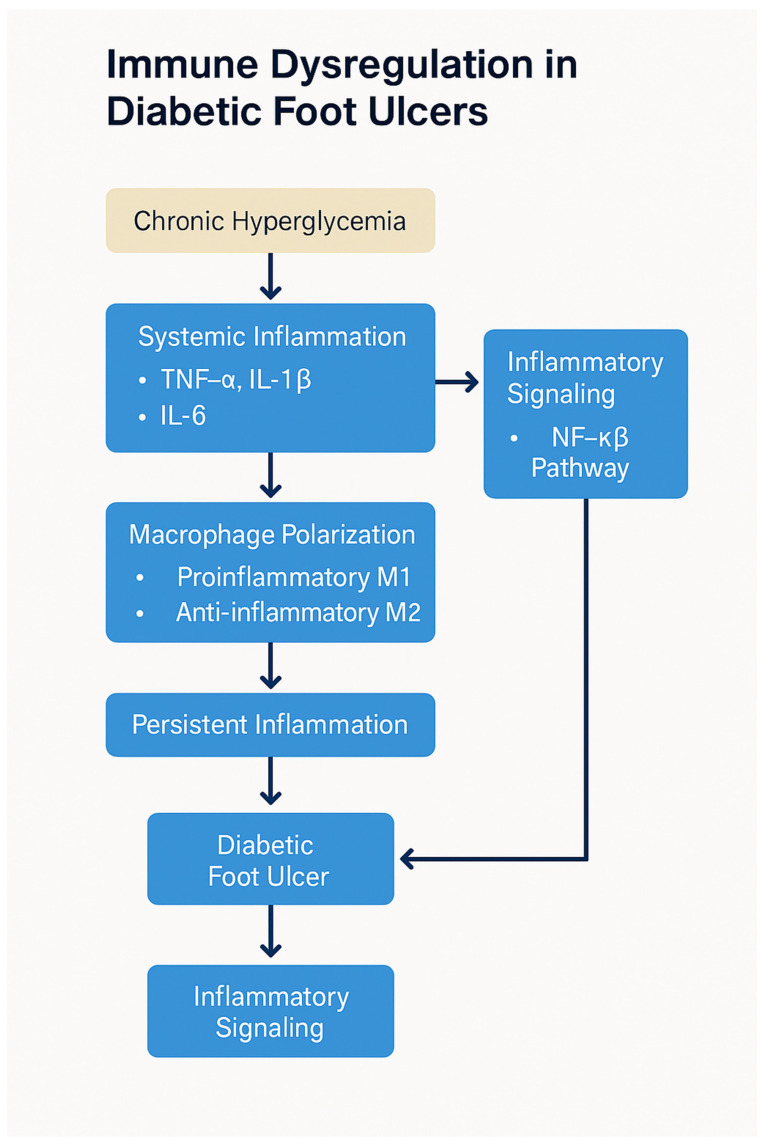
Immune dysregulation in diabetic foot ulcers. Chronic hyperglycemia triggers oxidative stress and AGE–RAGE signaling, leading to polarization of macrophages toward the pro-inflammatory M1 phenotype, depletion of regulatory T cells (Tregs), and impaired neutrophil function. These immune abnormalities sustain a chronic inflammatory environment, delay re-epithelialization, inhibit angiogenesis, and impair bacterial clearance, ultimately contributing to poor wound healing and DFU chronicity. Created by PowerPoint.

The immune dysfunction in diabetes not only hampers the healing of wounds but also increases susceptibility to infections. Neutrophils, which are critical for the early stages of infection control, are particularly impaired in diabetic individuals. Neutrophil dysfunction includes reduced chemotaxis, defective phagocytosis, and a diminished ability to release antimicrobial peptides, all of which contribute to an increased risk of infection in diabetic wounds. Furthermore, the impaired recruitment of immune cells to the wound site allows pathogens to proliferate, further compounding the problem [50]. The increased risk of infections, combined with the delayed immune response, creates a vicious cycle that impedes wound healing (Figure 3). Chronic infection exacerbates inflammation, further prolonging the healing process and increasing the risk of severe complications such as osteomyelitis or amputation. Additionally, infections lead to the release of systemic inflammatory mediators, which can impair organ function and further complicate diabetes management [50].

### 3.5. Therapeutic Strategies Targeting Immune Dysregulation

Given the central role of immune dysregulation in the delayed healing of DFUs, therapeutic approaches targeting specific immune pathways have been explored. Biologics that inhibit key pro-inflammatory cytokines, such as TNF-α, IL-1β, and IL-6, have shown promise in reducing inflammation and improving healing outcomes. These biologics, including monoclonal antibodies and soluble receptor inhibitors, are currently being tested in clinical trials for their efficacy in managing chronic wounds in diabetic patients [51].

Another therapeutic strategy is to promote macrophage polarization toward the reparative M2 phenotype. This can be achieved through the use of agents that block the signaling pathways driving M1 polarization, such as NF-κB or MAPK inhibitors, or by introducing growth factors like TGF-β that promote M2 macrophage differentiation. In addition, enhancing angiogenesis through targeted therapies that increase VEGF expression or using stem cell-based therapies to promote vascularization is also under investigation [51]. Restoring the proper function of T cells, particularly regulatory T cells, also holds therapeutic potential. Strategies that boost Treg function or increase their numbers at the wound site could help reduce chronic inflammation and facilitate tissue repair, accelerating the healing process in diabetic foot ulcers [52].

Recent studies have identified microRNAs (miRNAs) as key post-transcriptional regulators involved in immune modulation and wound healing in diabetic foot ulcers [53]. miR-146a, for instance, targets critical components of the Toll-like receptor signaling pathway, including IRAK1 and TRAF6, thereby attenuating NF-κB activation and reducing the production of pro-inflammatory cytokines such as TNF-α and IL-6 [54]. In diabetic wounds, miR-146a expression is often dysregulated, contributing to persistent inflammation and impaired healing [54].

Similarly, miR-132 plays an important role in resolving inflammation and promoting the proliferative phase of wound healing [55]. It suppresses NF-κB signaling and reduces the expression of inflammatory chemokines, including IL-8, CXCL1, and CXCL5 in keratinocytes [55]. Inhibition of miR-132 in diabetic models has been associated with delayed wound closure and impaired re-epithelialization, suggesting a therapeutic role for miR-132 mimics [55].

miR-210 has also emerged as a key regulator of cellular responses to hypoxia, a critical component of chronic wound environments such as DFUs [56]. Studies have shown that miR-210 levels are inversely correlated with hypoxia-inducible factor-1α (HIF-1α) in DFU tissues, implying a role in angiogenic signaling and tissue oxygenation [56].

In addition, miR-126-3p promotes angiogenesis and endothelial cell migration by activating pro-survival signaling pathways such as Akt and ERK1/2 [57]. Overexpression of miR-126-3p in diabetic mouse models has been shown to enhance vascularization and accelerate wound healing [57].

These findings suggest that therapeutic modulation of miRNAs using mimics or inhibitors (antagomirs) may represent a promising approach to restoring immune balance and promoting wound repair in diabetic foot ulcers [14]. Future strategies may also involve the use of exosome-based delivery systems enriched with therapeutic miRNAs to target wound environments with precision [14].

Incorporating immune-modulatory nanomaterials and bioengineered scaffolds into wound dressings is also being investigated for their potential to deliver cytokines, promote immune cell recruitment, and resolve inflammation. These platforms can be engineered to release IL-10, VEGF, or antimicrobial peptides in a controlled fashion, while simultaneously supporting tissue regeneration through enhanced fibroblast migration and angiogenesis [12].

## 4. Current and Emerging Therapeutic Strategies for DFUs

The management of diabetic foot ulcers (DFUs) requires an integrated, multidisciplinary approach that addresses both systemic metabolic dysfunction and localized wound healing processes. Current therapeutic strategies for DFUs emphasize optimizing glycemic control, preventing and treating infection, and implementing advanced wound care techniques. However, recent advancements in immunomodulatory and regenerative medicine therapies are showing promise in improving healing outcomes and reducing ulcer recurrence [12].

### 4.1. Glycemic Control

Glycemic control remains the cornerstone of DFU management, as persistent hyperglycemia significantly impairs wound healing by promoting a chronic inflammatory state, reducing angiogenesis, and increasing susceptibility to infection. Clinical trials have demonstrated that tight glycemic control achieved through insulin therapy or oral hypoglycemic agents, such as metformin, sulfonylureas, and SGLT2 inhibitors, improves wound healing and reduces amputation rates in patients with DFUs [58,59]. In particular, SGLT2 inhibitors have shown additional benefits beyond glucose reduction, such as decreasing oxidative stress and inflammation, which may enhance tissue repair [15,59]. Additionally, real-world studies have shown that patients on SGLT2 inhibitors experience fewer diabetic foot-related hospitalizations, potentially due to reduced endothelial damage and improved microvascular function. Studies show that SGLT2 inhibitors enhance nitric oxide bioavailability, attenuate AGE–RAGE signaling, and improve wound perfusion in diabetic models [39].

- **Insulin:** Dosage is individualized based on blood glucose monitoring and patient needs. Adjustments are made to achieve target glycemic levels while minimizing hypoglycemia. When initiating SGLT2 inhibitors in patients already on insulin with HbA1c < 58 mmol/mol and eGFR > 45 mL/min/1.73 m^2^, consider reducing the insulin dose by 20% to avoid hypoglycemia [59].
- **Metformin:** The typical starting dose is 500 mg orally once or twice daily with meals, gradually increasing to a maximum of 2000–2500 mg daily, divided into multiple doses. It is recommended for patients with an estimated glomerular filtration rate (eGFR) ≥ 30 mL/min/1.73 m^2^ [59].
- **SGLT2 Inhibitors:**
  ○ **Dapagliflozin:** 10 mg once daily.
  ○ **Empagliflozin:** 10 mg once daily, which may be increased to 25 mg daily.
  ○ **Canagliflozin:** 100 mg once daily, which may be increased to 300 mg daily. These medications are typically initiated in patients with eGFR ≥ 20 mL/min/1.73 m^2^ and continued as tolerated until dialysis or transplantation is initiated [59].

Recent preclinical work has demonstrated that SGLT2 inhibitors not only lower glucose but may also influence immune cell metabolism. Specifically, empagliflozin has been shown to reduce neutrophil extracellular trap (NET) formation [60], suggesting that these drugs may offer dual metabolic and immunologic benefits in DFUs.

### 4.2. Wound Care and Infection Control

Effective wound care is crucial for preventing infection and promoting tissue regeneration in DFUs. Standard wound care includes debridement, infection control, and offloading pressure from the affected foot.

○ **Debridement** involves the removal of necrotic tissue to prevent bacterial colonization and facilitate new tissue growth. Enzymatic, autolytic, and sharp surgical debridement methods are employed based on the wound’s characteristics [59].
○ **Infection control** is vital, as infected DFUs significantly increase the risk of limb amputation. Empirical antibiotic therapy targeting common pathogens such as *Staphylococcus aureus* and *Pseudomonas aeruginosa* is initiated while awaiting culture results. Multidrug-resistant infections often require combination antibiotic therapy and, in severe cases, surgical intervention [59]. Recent consensus guidelines also recommend early use of biofilm-disrupting agents in chronic DFUs, as biofilms are highly prevalent and contribute to antibiotic resistance. Agents such as cadexomer iodine [61] and polyhexamethylene biguanide (PHMB) [62] have shown efficacy in reducing bacterial load and promoting granulation tissue formation.
○ **Offloading techniques**, such as total contact casting (TCC) and removable cast walkers, are employed to reduce pressure and mechanical stress on the ulcer, thus promoting healing [59].

### 4.3. Advanced Wound Care Therapies

Emerging wound care strategies aim to enhance tissue regeneration through bioactive materials and cellular therapies:

○ **Bioactive dressings**, such as hydrocolloids, hydrogels, and antimicrobial silver dressings, provide a moist wound environment while reducing bacterial load, which accelerates healing [15]. Additionally, electroceutical dressings that generate low-intensity electrical fields have demonstrated antimicrobial activity and enhanced epithelial migration in DFUs. These devices may reduce bacterial biofilm adherence and improve oxygenation, representing a promising adjunct to traditional dressings [13].
○ **Growth factor therapy** involves the application of recombinant platelet-derived growth factors (e.g., becaplermin), which stimulate fibroblast proliferation and angiogenesis [59].
○ **Negative pressure wound therapy (NPWT),** or vacuum-assisted closure, enhances wound healing by promoting granulation tissue formation and removing excess wound exudate, thereby reducing infection risk [44,59].

### 4.4. Immunomodulatory and Anti-Inflammatory Therapies

Recent research highlights the role of chronic inflammation and immune dysregulation in DFU pathogenesis (Table 1). Several immunomodulatory approaches are under investigation:

○ **SGLT2 inhibitors**, traditionally used for glycemic control, have demonstrated anti-inflammatory effects by reducing pro-inflammatory cytokines, such as TNF-α and IL-6, potentially accelerating wound healing [15].
○ **Biologics**, such as TNF-α inhibitors (e.g., infliximab) and IL-1 receptor antagonists (e.g., anakinra), are being explored for their ability to modulate excessive inflammation and enhance immune function in chronic DFUs [59].
○ **Infliximab**: Administered as an intravenous infusion, typically starting with 5 mg/kg at weeks 0, 2, and 6, followed by maintenance doses every 8 weeks. Dosage adjustments may be necessary based on clinical response and tolerability.
○ **Anakinra**: Administered as a subcutaneous injection of 100 mg daily. The duration of therapy varies based on clinical response and physician discretion. Other promising agents include IL-17 inhibitors, such as secukinumab, which have shown preliminary success in modulating skin inflammation and may hold potential for future DFU trials, especially in patients with psoriatic or inflammatory comorbidities [11].

### 4.5. Regenerative Medicine and Gene Therapy

Regenerative approaches offer novel avenues for DFU treatment by enhancing tissue repair and neovascularization:

○ **Stem cell-based therapies**, including mesenchymal stem cells (MSCs) and induced pluripotent stem cells (iPSCs), have demonstrated the potential to differentiate into endothelial cells and fibroblasts, promoting tissue regeneration and angiogenesis in chronic wounds [24]. Allogeneic MSCs derived from adipose or umbilical sources have shown immunomodulatory properties, such as IL-10 secretion and inhibition of M1 macrophages, making them attractive for treating chronic non-healing ulcers [67].
○ **Gene therapy**, targeting angiogenic factors such as vascular endothelial growth factor (VEGF), aims to enhance local blood flow and accelerate wound healing. Preclinical studies have demonstrated promising results, but further research is needed for clinical application [68].

By integrating these strategies, clinicians can optimize DFU management and improve patient outcomes. Ongoing research into immune modulation and regenerative medicine continues to expand the therapeutic landscape, offering hope for more effective and personalized treatment approaches

## 5. Methods

A comprehensive literature search was conducted using PubMed for articles published between January 2010 and February 2024. The search employed combinations of terms, including “diabetic foot ulcer”, “immune dysregulation”, “macrophage polarization”, “regenerative therapy”, and “stem cells”. The inclusion criteria focused on human studies, reviews, and clinical trials relevant to DFU pathophysiology, immune mechanisms, and emerging therapies. The exclusion criteria included animal-only studies, articles lacking original data, and publications unrelated to diabetic wound healing or immunology. After screening 112 titles and abstracts, 35 articles were excluded for irrelevance or duplication. A total of 77 articles met the inclusion criteria and were synthesized in this review (Table 2).

## 6. Conclusions

Diabetic foot ulcers (DFUs) remain one of the most complex and costly complications of diabetes, driven by peripheral neuropathy, vascular dysfunction, metabolic imbalance, and chronic inflammation. Their progression from superficial lesions to deep infections and gangrene underscores the need for timely detection and comprehensive intervention. Although conventional strategies—such as glycemic control, infection management, debridement, and offloading—are foundational, they often prove insufficient due to delayed diagnosis, poor adherence, and disparities in healthcare access. Recent advances have illuminated the critical role of immune dysregulation in DFU pathogenesis. Impaired macrophage polarization, diminished regulatory T cell activity, and persistent pro-inflammatory signaling create a hostile wound environment that impedes healing. Immunomodulatory therapies—including biologics targeting TNF-α and IL-1β, Treg-based interventions, and novel anti-inflammatory agents—offer new therapeutic possibilities by restoring immune balance and promoting tissue repair. Regenerative approaches, such as stem cell-based therapies, gene delivery of angiogenic factors, and bioengineered scaffolds, provide complementary tools to enhance revascularization and tissue regeneration. Emerging technologies like artificial intelligence and wearable devices may further support early detection and guide real-time, personalized wound care. Looking forward, effective DFU management will require the integration of immune-targeted therapies within precision medicine frameworks and multidisciplinary care models. Expanding access to advanced therapies—particularly in underserved populations—remains a public health priority. Bridging basic science, clinical research, and digital health innovation will be essential for advancing equitable, personalized, and durable solutions for DFUs.

## Figures and Tables

**Table 1:** Summary of DFU treatment options.

Treatment Category	Specific Interventions	Mechanism of Action	Dosage
**Glycemic Control**	Insulin, Metformin, SGLT2 inhibitors	Reduces hyperglycemia, oxidative stress, and inflammation [42,44]	Individualized insulin dosing; Metformin 500–2500 mg/day; SGLT2 inhibitors (Dapagliflozin 10 mg/day, Empagliflozin 10–25 mg/day, Canagliflozin 100–300 mg/day) [45]
**Wound Care**	Debridement, Offloading, Antibiotic therapy	Removes necrotic tissue, reduces infection risk, and alleviates pressure [43]	Empirical antibiotics per IDSA guidelines (e.g., cephalexin 500 mg q6h, clindamycin 300 mg q8h) [63,64]
**Advanced Wound Therapies**	Bioactive dressings, Growth factors, NPWT	Enhances moisture balance, promotes angiogenesis, and accelerates healing [43,44,46]	Becaplermin 0.01% gel once daily; NPWT at 125 mmHg for 4–7 days [65,66]
**Immunomodulatory Therapies**	SGLT2 inhibitors, TNF-α inhibitors, IL-1 antagonists	Reduces inflammation and improves immune response [44,46]	Infliximab 5 mg/kg IV at weeks 0, 2, 6, then every 8 weeks; Anakinra 100 mg SC daily
**Regenerative Medicine**	Stem cell therapy (MSCs, iPSCs), Gene therapy (VEGF)	Stimulates tissue repair and angiogenesis [47]	N/A

N/A—not applicable.

**Table 2:** Summary table.

Stage	Articles
Identified through PubMed (2010–2024)	112
Excluded (irrelevant, duplicates, animal-only)	35
Included in this review	77

## Data Availability

Data are contained within the article.
